# Cortical activation change induced by neuromuscular electrical stimulation during hand movements: a functional NIRS study

**DOI:** 10.1186/1743-0003-11-29

**Published:** 2014-03-05

**Authors:** Sung Ho Jang, Woo Hyuk Jang, Pyung Hun Chang, Seung-Hyun Lee, Sang-Hyun Jin, Young Gi Kim, Sang Seok Yeo

**Affiliations:** 1 Department of Physical Medicine and Rehabilitation, College of Medicine, Yeungnam University, Daegu, Republic of Korea; 2 Graduate School, Daegu Gyeongbuk Institute of Science & Technology, Daegu, Republic of Korea; 3 Robot System Research Division, Daegu Gyeongbuk Institute of Science & Technology, Daegu, Republic of Korea; 4 Leaders rehabilitation hospital, Daegu, Republic of Korea; 5 Department of Physical Therapy, College of Health Sciences, Dankook University, 119, Dandae-ro, Dongnam-gu, Cheonan-si, Chungnam 330-714, Republic of Korea

**Keywords:** Neuromuscular electrical stimulation, Functional near infrared spectroscopy, Motor learning, Cortical activation

## Abstract

**Objectives:**

Neuromuscular electrical stimulation (NMES) has been used in the field of rehabilitation for a long time. Previous studies on NMES have focused on the peripheral effect, in contrast, relatively little is known about the effect on the cerebral cortex. In the current study, we attempted to investigate the change of cortical activation pattern induced by NMES during execution of hand movements in normal subjects, using functional near infrared spectroscopy (fNIRS).

**Methods:**

Twelve healthy normal subjects were randomly assigned to the NMES group (six subjects) and the sham group (six subjects). We measured oxy-hemoglobin (HbO) in six regions of interest (ROI) during pre-NMES and post-NMES motor phase; the left dorsolateral and ventrolateral prefrontal cortex, premotor cortex, primary sensory-motor cortex (SM1), hand somatotopic area of SM1, and posterior parietal cortex. Between the pre-NMES and the post-NMES motor phases, real or sham NMES was applied on finger and wrist extensors of all subjects during a period of 5 minutes.

**Results:**

In all groups, during the pre-NMES motor phase, the HbO value in the hand somatotopic area of the left SM1 was higher than those of other ROIs. In the NMES group, during the post-NMES motor phase, HbO value variation in the hand somatotopic area of the left SM1 showed a significant decrease, compared with that of sham group (*p* < 0.05). However, in the sham group, similar aspect of results in HbO values of all ROIs was observed between pre-NMES and post-NMES motor phases (*p* > 0.05).

**Conclusions:**

Results of this study showed that NMES induced a decrease of cortical activation during execution of hand movements. This finding appears to indicate that application of NMES can increase the efficiency of the cerebral cortex during execution of motor tasks.

## Introduction

Neuromuscular electrical stimulation (NMES) has been used in the field of rehabilitation for a long time [1-8]. NMES induces contraction of the underlying muscles by application of electrical current [1-8]. Many studies have reported on the effect of NMES in prevention of muscle atrophy, decrease of spasticity, increase of muscle strength, and facilitation of recovery of functional movement [2,4,7,9-15]. In addition, several studies have reported that NMES has a direct effect on the cortical activation [16-19]. On the other hand, some studies have reported changes of cortical activation with functional recovery of the hemiparetic hand after long-term intensive treatment by NEMS [20,21]. However, little is known about the short-term effect on the changes of cortical activation during motor performance after application of NMES. In this study, we hypothesized that NMES might affect the cortical activation during execution of active movements.

Several functional neuroimaging techniques, such as functional MRI, magnetoencephalography, Positron Emission Tomography, and functional near infrared spectroscopy (fNIRS) are available for use in studies of brain activation induced by NMES [18,20-23]. Among these techniques, fNIRS, a new emerging distinguished optical instrument, based on the intrinsic optical absorption of blood, enables noninvasive measurement of regional relative hemodynamic responses associated with cortical activation [24-28]. Compared with other functional neuroimaging techniques, fNIRS system has certain advantages, including lower price and portability [27]. In particular, due to less sensitivity to electrical current and metallic effect of the NMES machine, fNIRS could be appropriate for research on brain activation patterns by NMES [17,20,24-26].

In the current study, using fNIRS, we attempted to investigate the change of cortical activation pattern induced by NMES during execution of hand movements in normal subjects.

## Materials and methods

### Subjects

Twelve healthy volunteers (seven men, mean age: 29.00 ± 3.25 years) with no history of neurological, psychiatric, or physical illness were enrolled in this study. They were randomly assigned to the NMES (six subjects, four men, mean age: 27.17 ± 3.43 years) and sham groups (six subjects, three men, mean age: 30.83 ± 1.83 years). All participants were right-handed as verified by the modified Edinburgh Handedness Inventory [29]. They gave written informed consent prior to participation in this study. The study protocol was approved by the institutional review board in Yeungnam university medical center (YUH-12-0419-D12).

### Experimental design

**Figure 1:**
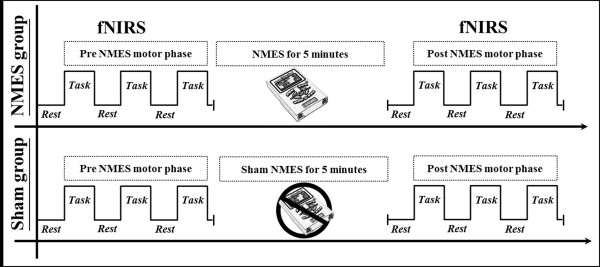
**The paradigm of functional near infrared spectroscopy (fNIRS) scanning.** The fNIRS paradigm consisted of two consecutive phases, the pre-neuromuscular electrical stimulation (NMES) motor phase and the post-NMES motor phase. Between the pre-NMES and the post-NMES motor phase, electrical stimulation for the finger and wrist extension was provided to subjects in the NMES group for 5 minutes, and those in the sham electrical stimulation group for 5 minutes.

All subjects were asked to sit comfortably on a chair in an upright position during the experiment. NMES was applied through a 2-channel electrical stimulator (EMGFES 1000, Cyber Medic, Republic of Korea). Monophasic square wave pulses were used at the rate of 35 Hz with a pulse width of 250us, pulsed 3 seconds on and 2 seconds off. Extension movements of fingers and wrist were performed using one channel through round surface stimulation electrodes fixed to the skin with adhesive gel. The electrodes were positioned with a cathode over the extensor digitorum communis and an anode on the forearm near the wrist. The stimulation intensity was adjusted to produce the maximum extension of the fingers and wrist within the limit that the subject did not feel any discomfort (range of stimulation intensity: 10 ~ 15 mA).The fNIRS paradigm consisted of two consecutive phases, the pre-NMES motor phase and the post-NMES motor phase. Changes of oxy-hemoglobin (HbO) were measured during the pre-NMES motor phase and the post-NMES motor phase. Using a block paradigm design (three cycles; resting [20 sec] - hand movements [20 sec] - resting [20 sec] - hand movements [20 sec] –resting [20 sec] - hand movements [20 sec]), all subjects performed grasp-release movements of right hand under metronome guidance at a frequency of 0.5 Hz (Figure 1). Between the pre-NMES motor phase and the post-NMES motor phase, electrical stimulation for the finger and wrist extension movements was applied for 5 minutes.

### Functional NIRS

The continuous wave fNIRS system (FOIRE-3000; Shimadzu, Kyoto, Japan), with continuous wave laser diodes with wavelengths of 780, 805, and 830 nm, was used for recording of cortical activity at a sampling rate of 10 Hz; we employed a 49-channel system with 30 optodes (15 light sources and 15 detectors). Based on the modified Beer–Lambert law, we acquired values for oxy-hemoglobin (HbO) following changes in levels of cortical concentration [28,30]. The international 10/20 system, with Cz (cranial vertex) located beneath the 18th channel, between the fourth light source and the seventh detector, was used for positioning of optodes; locations of the nasion, left ear and right ear were measured in each subject. A stand-alone application was used for spatial registration of the acquired 49 channels on the Montreal Neurological Institute (MNI) brain based on locations of the nasion, left ear and right ear, and the 18th channel on the Cz [28].

The software package NIRS-SPM (Near Infrared Spectroscopy-Statistical Parametric Mapping) (http://bisp.kaist.ac.kr/NIRS-SPM) implemented in the MATLAB environment (The Mathworks, USA) was used in analysis of fNIRS data. Gaussian smoothing with a full width at half maximum (FWHM) of 2 s was applied to correction of noise from the fNIRS system [30]. The wavelet-MDL based detrending algorithm was used for correction of signal distortion due to breathing or movement of the subject and then general linear model (GLM) analysis with canonical hemodynamic response curve to model the hypothesized HbO response during experimental condition was performed [28]. SPM t-statistic maps were computed for group analysis, and HbO was considered significant at an uncorrected threshold of *p* < 0.01 for stricter analysis.

**Figure 2:**
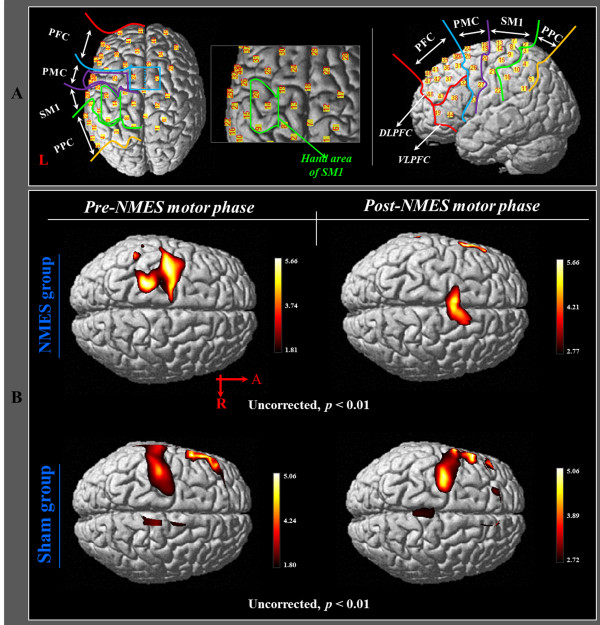
**The results of group analysis of HbO in NMES group and sham group. (A)** Six regions of interest based on the anatomical location of the brain. **(B)** Group-average activation map of HbO during pre NMES motor phase and post NMES motor phase using NIRS-SPM (uncorrected, *p* < 0.01).

In order to investigate the cortical changes of HbO during execution of hand movements, we selected six regions of interest (ROI) based on the Brodmann area (BA) and anatomical locations of brain areas: primary sensory-motor cortex (SM1) (BA 1, 2, 3, and 4), the hand somatotopic area of the SM1 (medial boundary: medial margin of the precentral knob, lateral boundary: lateral margin of the precentral knob), premotor cortex (PMC) (BA 6), dorsolateral prefrontal cortex (DLPFC)(BA 8,9,46), ventrolateral prefrontal cortex (VLPFC)(BA 44,45,47), and posterior parietal cortex (PPC) (BA 5,7) (Figure 2-A) [31-33]. Values for regional changes of HbO were estimated during the task phases of right hand movement from each channel of six ROIs; HbO values of each ROI were acquired based on the individual GLM analysis results, and we calculated HbO value variation at the post-NMES motor phase based on the result of the HbO value at the pre-NMES motor phase.

### Statistical analysis

SPSS software (v.15.0; SPSS, Chicago, IL) was used for data analysis. Mann-Whitney test was used for determination of differences in HbO values variation at the post-NMES motor phase between NMES group and sham group. Results were considered significant when *p* value was < 0.05.

## Results

**Figure 3:**
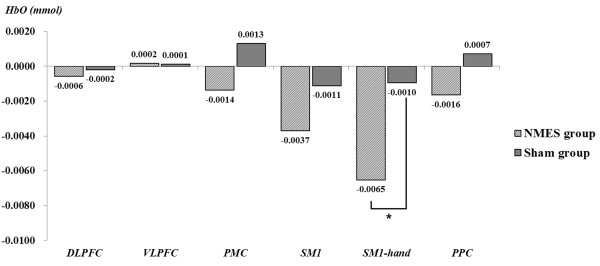
**The HbO value variation between pre-NMES motor phase and post-NMES motor phase in the NMES group and sham group.** In the NMES group, during the post-NMES motor phase, HbO value variation in the hand somatotopic area of the left SM1 was significantly decreased, compared with that of the sham group. *p < 0.05.

**Figure 4:**
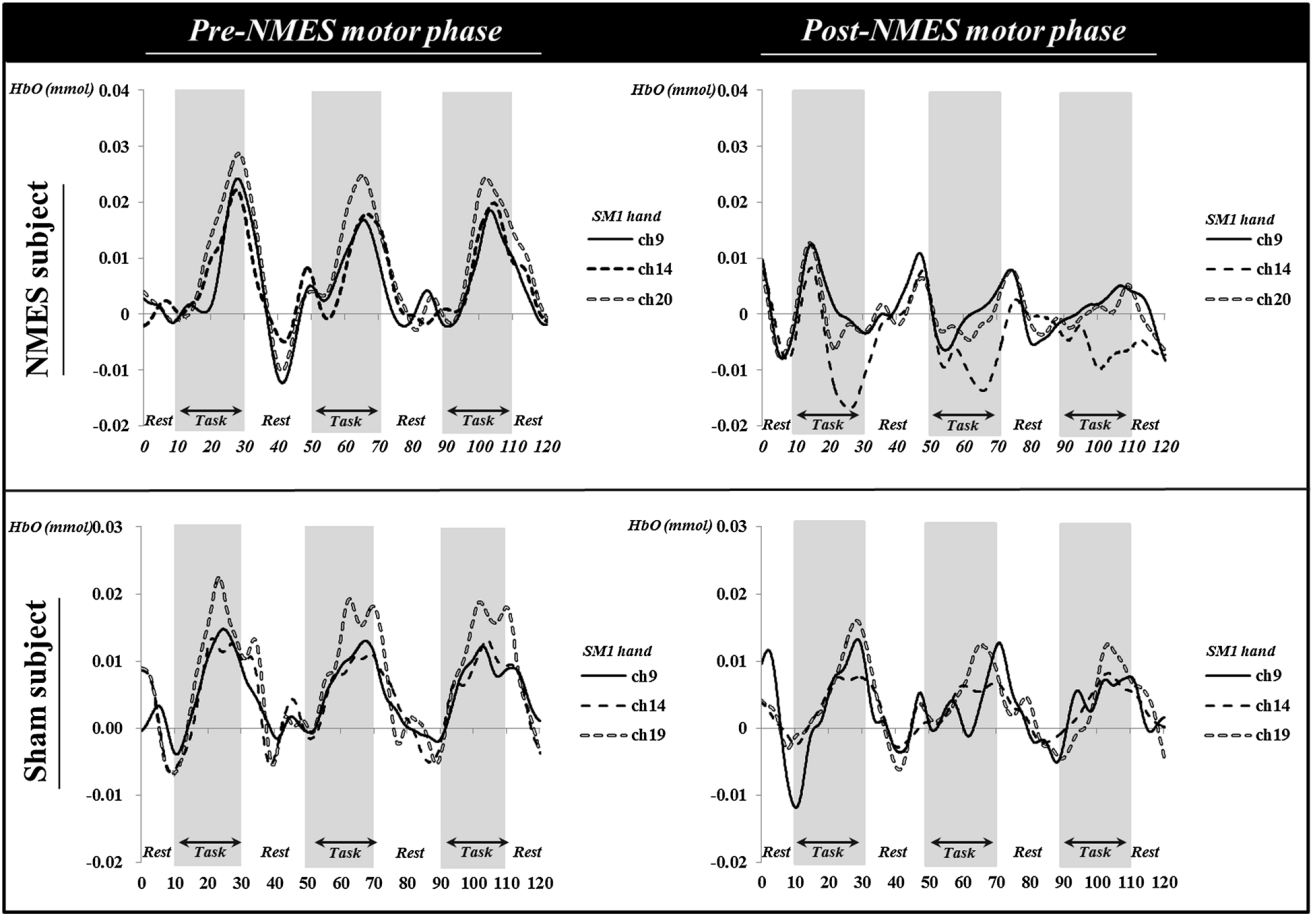
The different of time course of hemodynamic responses for oxy-hemoglobin (HbO) in the channels on the hand somatotopic area of the somatosensory motor cortex (SM1) between pre-neuromuscular electrical stimulation (NMES) motor phase and post-NMES motor phase (subject: a 28-year-old male; sham subject: a 28-year-old male).

In the NMES group, during the pre-NMES motor phase, the HbO value in the hand somatotopic area of the left SM1 was higher (HbO = 0.0082) than those of other ROIs (DLPFC: HbO = 0.0032; VLPFC: HbO = 0.0027; PMC: HbO = 0.0041; SM1: HbO = 0.0055; PPC: HbO = 0.0035). However, during the post-NMES motor phase, HbO value showed a significant decrease only in the hand somatotopic area of the left SM1 (HbO value variation = -0.0065), compared with that of HbO value variation (-0.0010) in the sham group (*p* < 0.05) (Figures 3 and 4). In addition, HbO value also decreased after NMES in the whole left SM1, however, no significant difference was observed (*p* > 0.05) (Figure 3).In the sham group, during the pre-NMES motor phase, the HbO value in the hand somatotopic area of the left SM1 was also higher (HbO = 0.0048) than those of other ROIs (DLPFC: HbO = 0.0028; VLPFC: HbO = 0.0033; PMC: HbO = 0.0015; SM1: HbO = 0.0045; PPC: HbO = 0.0021). However, similar results of HbO values in each ROI were observed during post-NMES motor phase (DLPFC: HbO = 0.0026; VLPFC: HbO = 0.0034; PMC: HbO = 0.0028; SM1: HbO = 0.0034; SM1 hand: HbO = 0.0039; PPC: HbO = 0.0028) (Figure 4).

Results of group analysis of HbO indicated significant activation of the left SM1 during pre-NMES motor phase in both groups (uncorrected, *p* < 0.01). In the post-NMES motor phase, the sham group showed similar activation in the SM1, compared with the pre-NMES motor phase (uncorrected, *p* < 0.01). In contrast, in the NMES group, no activation of HbO was observed in the left SM1 during the post-NMES motor phase (uncorrected, *p* < 0.01) (Figure 2-B).

## Discussion

In the current study, we investigated change in cortical activation during execution of grasp-release hand movements, following application of NMES on the finger and wrist extensors. We measured HbO as an index of neural activation. HbO, the most commonly used parameter of fNIRS, measures neural activity indirectly through detection of hemodynamic changes of the underlying cerebral cortex (oxygen consumption by neuronal cells) [26,34]. As a result, in the NMES group, we observed a significant decrease of cortical activation in the hand somatotopic area of the SM1, compared with that of the pre-NMES motor phase after application of NMES on finger and wrist extensors for 5 minutes. By contrast, no significant change was observed in the sham group. This result coincided with the results of t-statistic maps from HbO, indicating significant cortical activation of the hand somatotopic area in the SM1 during execution of grasp-release hand movements, and decreased cortical activation after NMES. Our results appear to suggest that application of appropriate electrical stimulation on the finger and wrist extensors might induce an increase in the efficiency of cortical activation of the hand somatotopic area in the SM1 during execution of hand movements.

We think that the most plausible physiological mechanism of our result showing that cortical activation by execution of hand movements was decreased after NMES appears to be related to the motor learning effect [35-43]. In detail, reduction of cortical activation of the hand somatotopic area in the SM1 after NMES in the NMES group might be caused by efficient processing in the cortical area by motor learning effect by NMES [35-41]. In addition, the result of the current study showed that this effect was achieved by short-term (5 minutes) intervention using NMES [42,43]. Many previous studies have suggested that passive movements by NMES were effective in improvement of motor control and functional abilities of the affected extremities in patients with brain injury through a motor learning effect [13-15,44-47]. However, this motor relearning effect by NMES has been acquired through long-term therapeutic periods [13-15,44-47]. With regard to the motor learning effect on cortical activation, many previous studies have suggested that the neural mechanisms underlying motor learning showed that recruitment of cortical neural resources increased in the early learning phase, and that cortical activities were decreased in the automatic phase [35-41]. By contrast, some studies have reported on reduction of cortical activation during the early motor learning phase [42,43]. In 1998, using sequential finger movements, Toni et al reported that decrease of activation in cortical areas following motor learning increased in normal subjects [43]. They reported that the PMC and supplementary motor area were highly activated in early learning phase, by contrast, the SM1 showed a continuous decrease in activation along with learning progress. In 2010, Park et al reported changes of cortical activities by continuous monitoring during the early motor learning period in normal subjects [42]. They reported that the SM1 activation increased by performance of finger motor tasks over a period of four minutes and then showed a continuous decrease with progress of the learning effect. The results of the two studies described above appear to be compatible with the results of the current study, which showed decreased cortical activation of the hand somatotopic area in the SM1 following NMES for 5 minutes. Therefore, it appears that appropriate electrical stimulation by application of NMES could induce a similar effect with motor training, which increases efficiency during short-term intervention.

In conclusion, we investigated the change of cortical activation by application of NMES during execution of hand movements in normal subjects and found that NMES induced a decrease of cortical activation during execution of motor tasks. This finding appears to indicate that application of NMES can increase the efficiency of the cerebral cortex during execution of motor tasks. To the best of our knowledge, this is the first study to demonstrate the effect of NMES on cortical activation during execution of motor tasks. We believe that the results of this study would be helpful for brain rehabilitation in relation to NMES. In addition, fNIRS is a valuable tool for use in research on the brain effect of NMES, which is employed in the field of brain rehabilitation. We believe that conduct of further studies on the long-term effect of NMES for brain activation and clinical trials for patients with brain injury is necessary. In addition, conduct of further studies on the optical conditions of NMES for cortical activation would be necessary.

## Competing interests

The authors declare that they have no competing interests.

## Authors’ contributions

SH Jang and SS Yeo conceived the project. SS Yeo, WH Chang, SH Lee and SH Jin performed experiments. SH Jang and SS Yeo data processing and analysis. SH Jang, PH Chang, YK Kim, and SS Yeo wrote the paper. All authors read and approved the final manuscript.
